# Single‐Cell RNA Sequencing Reveals Dynamic Expression Patterns of MAIT Cells and Macrophage Subpopulations in Myocardial Injury Model

**DOI:** 10.1155/ijog/5460709

**Published:** 2026-05-20

**Authors:** Haofei Kang, Junjie Wang, Renjie Ruan, Yudan Wen, Bingru Chen, Meixiang Wu, Yongguang Wang, Yunrui Zhang

**Affiliations:** ^1^ The First Ward of Cardiovascular Medicine, Yantaishan Hospital, No. 10087 Keji Road Laishan, Yantai, 264000, Shandong, China, ytsyy.com; ^2^ Department of Cardiology, The Third Affiliated Hospital of Wenzhou Medical University, No. 108 Wansong East Road, Rui’an, 325200, Zhejiang, China, wmu.edu.cn; ^3^ Internal Medicine Department, Tingtian Sub-District Community Health Service Center, No. 291 Wenhua Road Tingtian Street, Rui’an, 325200, Zhejiang, China

**Keywords:** IL-10, immune remodeling, macrophage polarization, MAIT cells, MR1, myocardial injury, single-cell RNA sequencing, TNF-α

## Abstract

**Background:**

Mucosal‐associated invariant T (MAIT) cells are innate‐like T lymphocytes involved in immune surveillance and tissue homeostasis. Yet, their transcriptional dynamics and functional crosstalk with macrophages at the time of myocardial injury are incompletely defined.

**Methods:**

We used two publicly available single‐cell RNA sequencing (scRNA‐seq) datasets from a murine ischemia–reperfusion myocardial injury model (GSM8252253 and GSM8252254). Following quality control and clustering, immune cell composition, cytokine expression patterns, and macrophage polarization states were systematically characterized. Concurrently, in vitro coculture experiments validated the MR1‐dependent regulatory effects of 5‐OP‐RU–activated MAIT cells on macrophage cytokine expression.

**Results:**

Single‐cell analysis showed significant immune remodeling on Day 7 post‐injury, with a relative expansion of B cells and depletion of macrophages compared to controls. UMAP‐based feature mapping revealed that expression of Tnf was significantly restricted to myeloid compartments (especially monocytes), whereas Il10 exhibited a diffuse pattern across immune subsets. MAIT cells exhibited increased expression of signature genes such as ZBTB16 and KLRB1 consistent with activation‐associated transcriptional reprogramming after damage. Macrophage populations exhibited heterogeneous polarization states, with limited TNF‐α/IL‐10 coexpression and overall low Il10 abundance in vivo. Functional validation showed that 5‐OP‐RU–activated MAIT cells significantly suppressed TNF‐α while enhancing IL‐10 expression in macrophages, and these effects were largely reversed by MR1 blockade.

**Conclusions:**

Our findings reveal dynamic immune cell remodeling and transcriptional adaptation of MAIT cells during myocardial injury. The predominance of Tnf‐driven myeloid signaling and the MR1‐dependent anti‐inflammatory modulation of macrophages by activated MAIT cells highlight a potential regulatory axis in cardiac inflammation. These results provide mechanistic insight into MAIT–macrophage interactions and suggest potential immunomodulatory targets for myocardial injury intervention.

## 1. Introduction

Myocardial injury induces intricate immune‐mediated​ inflammatory cascades involving a variety of immune cell populations that orchestrate both tissue damage and repair mechanisms [1]. Of these, mucosal‐associated invariant T (MAIT) cells have emerged as a significant regulators of tissue homeostasis and immune surveillance; however, their specific functions in cardiac pathophysiology are poorly understood [2, 3].

MAIT cells are a unique type of innate‐like T lymphocytes, with semi‐invariant T cell receptor (TCR) and rapid activation dynamics after recognition of microbial metabolites or inflammatory signals [2, 4]. These cells are characterized by the expression of a number of transcriptional regulators such as ZBTB16 (also known as PLZF) that directs their effector differentiation program and surface markers like KLRB1 (CD161) and IL18R1 which mediate their activation responses [5, 6]. The transcription factor ZBTB16 is especially important for MAIT cell development and functional maturation, dictating the expression levels of effector molecules and cytokine production capacity [5, 7].

The cardiac immune microenvironment after injury is shaped by a balance of the myriad resident and infiltrating immune populations, with macrophages being central orchestrators of inflammatory signals [8]. Macrophage functional polarization into the classically activated (M1) and alternatively activated (M2) states is a fundamental process governing tissue inflammation and repair [9, 10]. In general terms, M1 macrophages are defined by increased expression of Nos2 and proinflammatory cytokines such as TNF, driving antimicrobial responses and promotion of tissue clearance [9]. In contrast, M2 macrophages have markers like Mrc1 (mannose receptor) and Arg1 (arginase 1), facilitating processes of tissue remodeling and promoting healing [10].

Recent advances in single‐cell RNA sequencing (scRNA‐seq) technologies have transformed our understanding of cellular heterogeneity and functional states relevant to disease contexts [11]. This strategy allows for broad characterization of immune cell populations with unmatched resolution, uncovering previously unappreciated cellular subtypes and their gene expression programs [12]. Notably, in the context of myocardial injury, single‐cell analyses have started to delineate complexities of immune responses and pinpoint potential therapeutic targets [13, 14].

Working with publicly available single‐cell transcriptomic datasets, the present study provides a comprehensive characterization of MAIT cell populations and macrophage subsets in myocardial injury models, all for the presumption that you will get to cover up for gene expression patterns, changes in cellular composition, and distributions of functional markers. In support of these computational observations, our in vitro validation experiments showed that activated MAIT cells downregulate macrophage cytokine expression in an MR1‐dependent manner.

## 2. Methods

### 2.1. Data Acquisition and Source

The scRNA‐seq data used in this study were obtained from the Gene Expression Omnibus (GEO) database under the sample accession numbers GSM8252253 (normal, control group) and GSM8252254 (MId7Saline, myocardial injury day 7 with saline treatment, injury model group), which are stored as filtered feature‐bc matrix in h5 format and contain transcriptomic data from a murine ischemia–reperfusion myocardial injury model. The dataset includes high‐quality scRNA‐seq profiles of the normal control group (GSM8252253) and the myocardial injury treatment group (GSM8252254). The dataset encompasses samples from both control and treatment groups in myocardial injury models. The dataset includes transcriptomic information from cells under normal physiological conditions and following myocardial injury, providing fundamental data support for analyzing the molecular mechanisms of MAIT cells and other immune cell subsets during myocardial injury processes. All raw sequencing data underwent standardized processing to ensure data quality and comparability.

### 2.2. Data Preprocessing and Quality Control

All the obtained scRNA‐seq data underwent systematic quality control and preprocessing analyses in Seurat (Version 4.3.0) of R environment. Cell quality control was performed to initially select high‐quality cells based on the number of detected genes per cell, total transcript counts, and ratios of mitochondrial genes. More specifically, only cells with 200–5000 detected genes, less than 20% mitochondrial content, and < 10,000 UMI counts were kept. Quality control filtering was applied to eliminate poor‐quality cells and doublets. Gene expression data were then normalized by a combination of log2 transformation and scaling methods to remove the technical biases. The overall quality of the data was assessed by principal component analysis (PCA), and applied downstream analyses were confirmed as reliable. The accompanying analysis code has been deposited in a GitHub repository for reproducibility (link to be added upon acceptance: (https://github.com/ [to be added upon acceptance])).

### 2.3. Identification of Highly Variable Genes and Dimension Reduction

We used variance analysis methods to identify genes with high expression variability across cells, e.g., those that often contain meaningful biological information. Downstream analysis was performed on the top 2000 most variable genes. PCA, based on highly variable genes, was then conducted to summarize variation in the dataset. The first few principal components explaining most variance were selected to facilitate dimensionality reduction visualization, and a total of 30 principal components were calculated. A Uniform Manifold Approximation and Projection (UMAP) algorithm with default parameters was used to project high dimensional data into a 2‐d space for visualization and clustering analysis of cell populations.

### 2.4. Unsupervised Clustering and Cell Type Identification

Unsupervised clustering analysis was conducted based on the dimensionally reduced data, employing the Louvain algorithm (graph‐based clustering) to identify cell populations with similar transcriptional characteristics. A clustering resolution of 0.8 was applied to obtain suitable numbers of cell clusters. Differential expression gene analysis was performed for each cluster to identify cluster‐specific marker genes. Combined with known cell type marker gene expression patterns, each cluster was annotated for cell type identity, with particular attention to MAIT cell–related signature genes such as ZBTB16, SLC4A10, IL18R1, and KLRB1.

### 2.5. Functional Annotation and Analysis of Differential Expression

Genes that were significantly differentially expressed between normal control and myocardial injury groups were identified by comparison using nonparametric statistical methods incorporating the Wilcoxon rank‐sum test. To filter differential genes, log2 fold change and adjusted *p* values were set as the thresholds. Of particular interest, expression of immune‐related genes, such as cytokines, transcription factors, and surface receptors was also analyzed specifically for the MAIT cell populations. In addition, we assessed expression patterns of macrophage polarization markers (Mrc1, Arg1, and Nos2) associated with polarization under Th− conditions.

### 2.6. Cell Culture and Coculture Experiments

Murine macrophages (RAW264.7 cell line) and MAIT cells were purchased from the American Type Culture Collection (ATCC, Manassas, VA, USA). Macrophages were cultured in RPMI‐1640 medium (Gibco, USA) supplemented with 10% fetal bovine serum (FBS; Gibco, USA) and 1% penicillin–streptomycin at 37°C in a humidified incubator containing 5% CO_2_, while MAIT cells were maintained under the same conditions. For coculture experiments, macrophages were seeded in 6‐well plates and divided into four groups: control (macrophages cultured alone), MAIT_unstim (macrophages cocultured with resting MAIT cells), MAIT_5OPRU (macrophages cocultured with MAIT cells activated by 5‐(2‐oxopropylideneamino)‐6‐D‐ribitylaminouracil, 5‐OP‐RU, 100 nM), and MAIT_5OPRU_MR1block (macrophages cocultured with 5‐OP‐RU–activated MAIT cells in the presence of MR1 blocking antibody, 10 μg/mL; BioLegend, USA). After 24 h of coculture, macrophages were collected for subsequent RNA extraction and quantitative PCR analysis.

### 2.7. Quantitative Real‐Time PCR

Total RNA was extracted from macrophages using TRIzol reagent (Invitrogen, USA) according to the manufacturer’s protocol. RNA purity and concentration were determined spectrophotometrically, and 1 μg of total RNA was reverse‐transcribed into cDNA using the PrimeScript RT reagent kit (Takara, Japan). qPCR was performed with SYBR Green Master Mix (Applied Biosystems, USA) on a QuantStudio 5 real‐time PCR system (Thermo Fisher Scientific, USA). The target genes included tumor necrosis factor‐α (TNF‐α) and interleukin‐10 (IL‐10), with GAPDH serving as the internal reference. The amplification program was 95°C for 30 s, followed by 40 cycles of 95°C for 5 s and 60°C for 30 s. Primer sequences (5′ ⟶ 3′) were as follows: TNF‐α forward CCC​TCA​CAC​TCA​GAT​CAT​CTT​CT, reverse GCT​ACG​ACG​TGG​GCT​ACA​G; IL‐10 forward GCT​CTT​ACT​GAC​TGG​CAT​GAG, reverse CGC​AGC​TCT​AGG​AGC​ATG​TG; GAPDH forward AGG​TCG​GTG​TGA​ACG​GAT​TTG, reverse TGT​AGA​CCA​TGT​AGT​TGA​GGT​CA. Relative expression levels were calculated using the 2^−ΔΔCt^ method, with the control group serving as the calibrator.

### 2.8. Statistical Analysis and Data Visualization

All statistical analyses were performed using R software and related bioinformatics packages. Various visualization methods including violin plots, heatmaps, and scatter plots were employed to display gene expression patterns and cell population distribution characteristics. Stacked bar charts were used to compare relative abundance changes of different cell subsets under various experimental conditions. All analysis results underwent multiple comparison correction to ensure statistical significance reliability. For in vitro experiments, one‐way analysis of variance (ANOVA) was performed followed by Tukey’s post hoc test. Data are presented as mean ± standard deviation (SD), and *p* values < 0.05 were considered statistically significant.

## 3. Results

### 3.1. scRNA‐seq Data Quality Control and Filtering

Quality control metrics demonstrated comparable overall sequencing quality between the normal and MI day 7 (MId7Saline) samples. Density distributions of detected genes per cell and total UMI counts showed broadly overlapping profiles across conditions, indicating similar library complexity and capture efficiency (Figures 1(a) and 1(b)). Mitochondrial gene percentages were largely concentrated at low values in both samples, with the applied 20% threshold removing only a small fraction of potential low‐quality cells (Figure 1(c)). Scatter plots of UMI counts versus detected genes further supported expected positive relationships without prominent doublet‐like outliers, and mitochondrial content did not show pervasive elevation across the retained cells (Figures 1(d) and 1(e)). After filtering, 11,964 cells were retained in the normal sample and 13,393 cells in the MI day 7 sample, providing adequate cell numbers for downstream analyses (Figure 1(f)).

FIGURE 1Quality control and basic metrics of single‐cell RNA sequencing data. (a) Density distribution of detected gene numbers (nFeature_RNA) per cell in normal and MI day 7 (MId7Saline) samples. (b) Density distribution of total UMI counts (nCount_RNA) per cell across conditions. (c) Distribution of mitochondrial gene percentage (MT%) in each sample. The red dashed line indicates the 20% cutoff threshold used for quality filtering. (d) Scatter plot of total UMI counts versus detected gene numbers in the normal sample, colored by mitochondrial gene percentage. (e) Scatter plot of total UMI counts versus detected gene numbers in the MI day 7 sample. (f) Bar plot showing the number of high‐quality cells retained after filtering in each sample (normal: 11,964 cells; MId7Saline: 13,393 cells).

**Figure:**
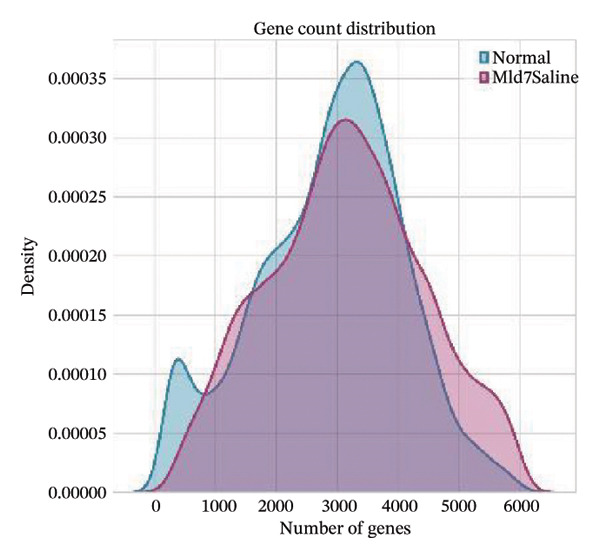
(a)

**Figure:**
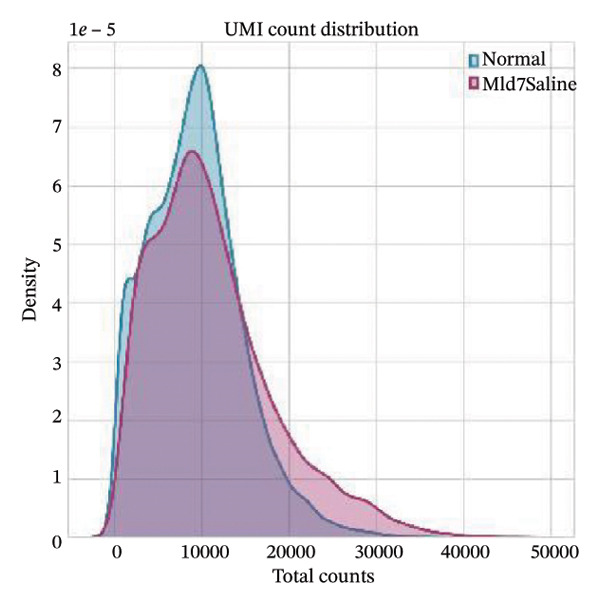
(b)

**Figure:**
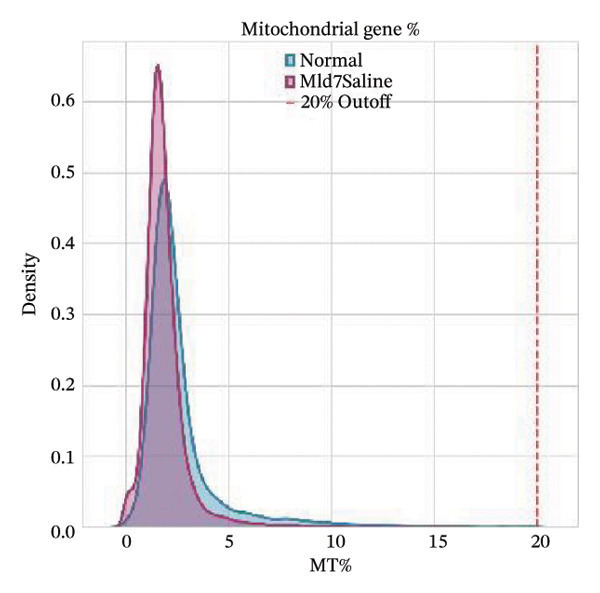
(c)

**Figure:**
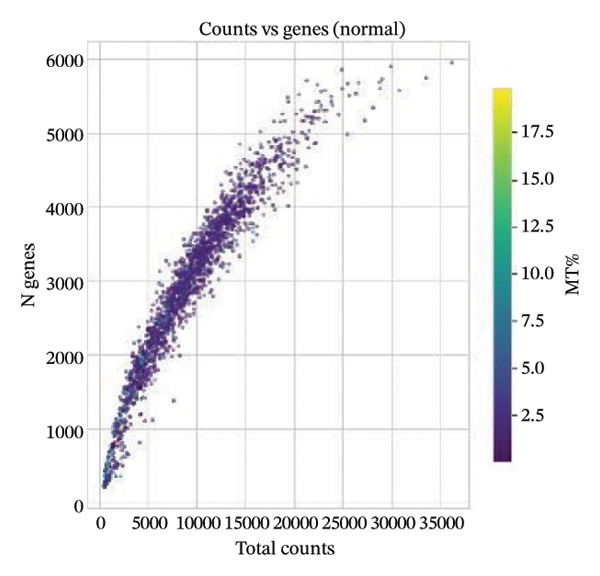
(d)

**Figure:**
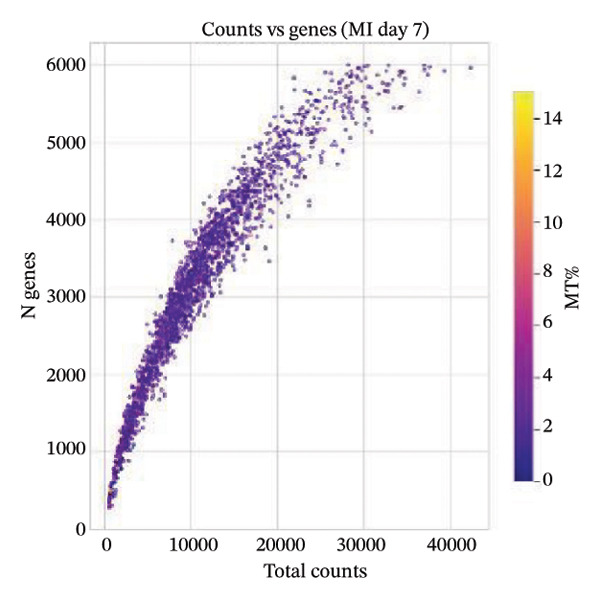
(e)

**Figure:**
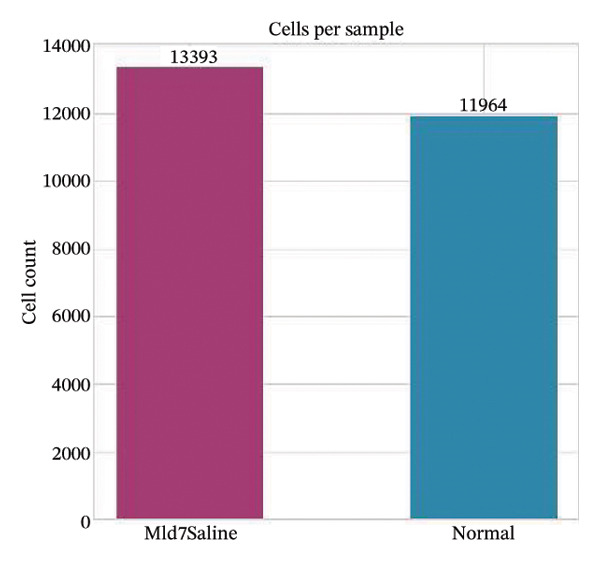
(f)

### 3.2. Dimensionality Reduction Shows Structured Cellular Heterogeneity and Strong Clustering

PCA showed that the first few components contained the largest sources of transcriptional variation in the dataset and thus justified graph‐based clustering (Figure 2(a)). In PCA space, subsets of cells from normal and MI day 7 showed partial overlap yet were displaced into condition‐associated directions (Figure 2(b)), consistent with remodeling associated with injury over a shared baseline structure (Figure 2(b)). Graph‐based Leiden clustering identified multiple transcriptionally distinct clusters, which were clearly separated on UMAP embeddings (Figure 2(c)). When colored by sample origin, both conditions were distributed across many clusters with regionally enriched densities, consistent with condition‐dependent changes in cellular composition and/or state (Figure 2(d)). Marker‐based annotation resolved major immune and cardiac/stromal compartments, including T cells, B cells, macrophages, monocytes, mast cells, cardiomyocytes, endothelial cells, smooth muscle cells, fibroblasts, and other immune cells (Figure 2(e)). Cluster composition analysis further indicated uneven contributions of clusters across conditions, supporting downstream cell type–stratified comparisons (Figure 2(f)).

FIGURE 2Dimensionality reduction, clustering, and cell type annotation of cardiac single‐cell transcriptomes. (a) Principal component analysis (PCA) showing the variance explained by the top 20 principal components. The red line indicates cumulative variance explained. (b) PCA plot colored by sample origin (normal vs. MId7Saline), illustrating global transcriptional similarity and separation. (c) UMAP visualization of Leiden clusters identified using graph‐based clustering (resolution = 0.8). (d) UMAP plot colored by sample origin, demonstrating distribution of cells from normal and MI day 7 samples. (e) UMAP plot annotated with major cell types based on canonical marker gene expression, including T cells, B cells, macrophages, monocytes, mast cells, cardiomyocytes, endothelial cells, smooth muscle cells, fibroblasts, and other immune cells. (f) Heatmap showing cluster composition percentages across normal and MId7Saline samples.

**Figure:**
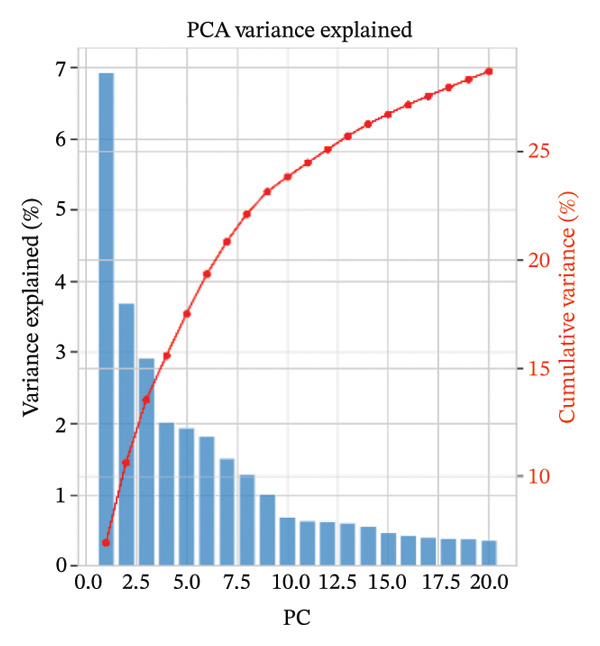
(a)

**Figure:**
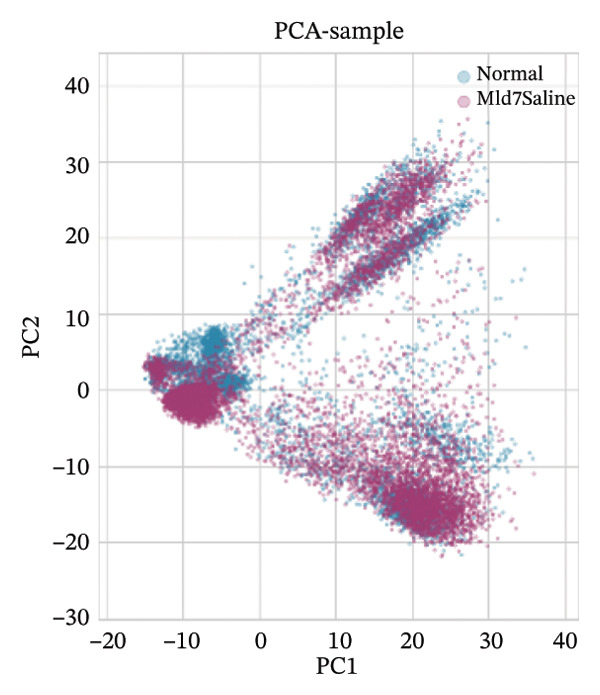
(b)

**Figure:**
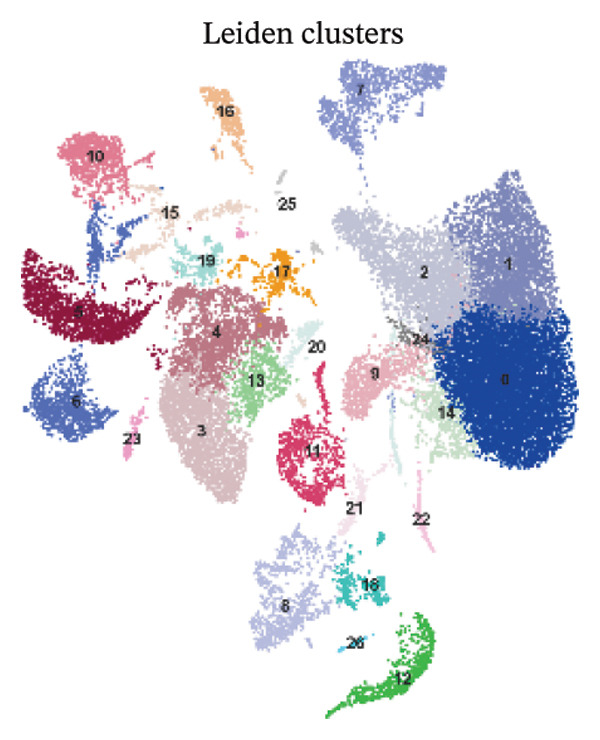
(c)

**Figure:**
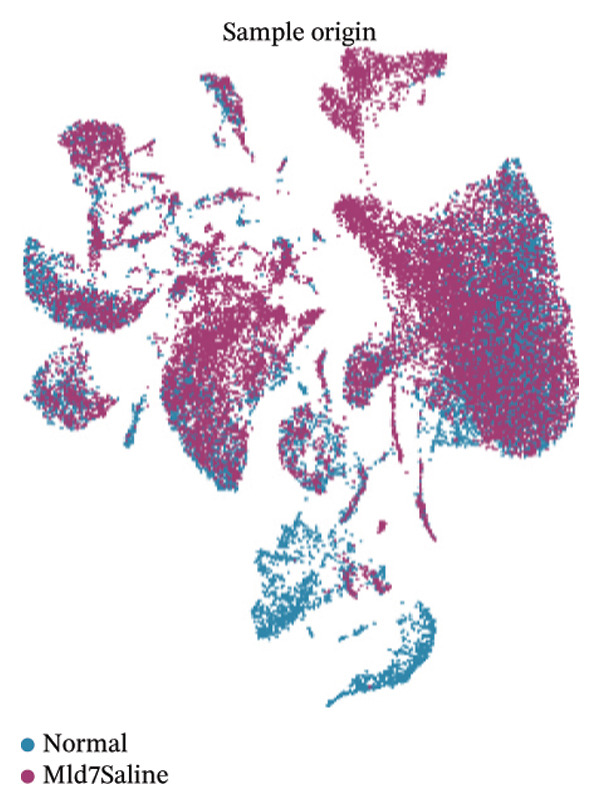
(d)

**Figure:**
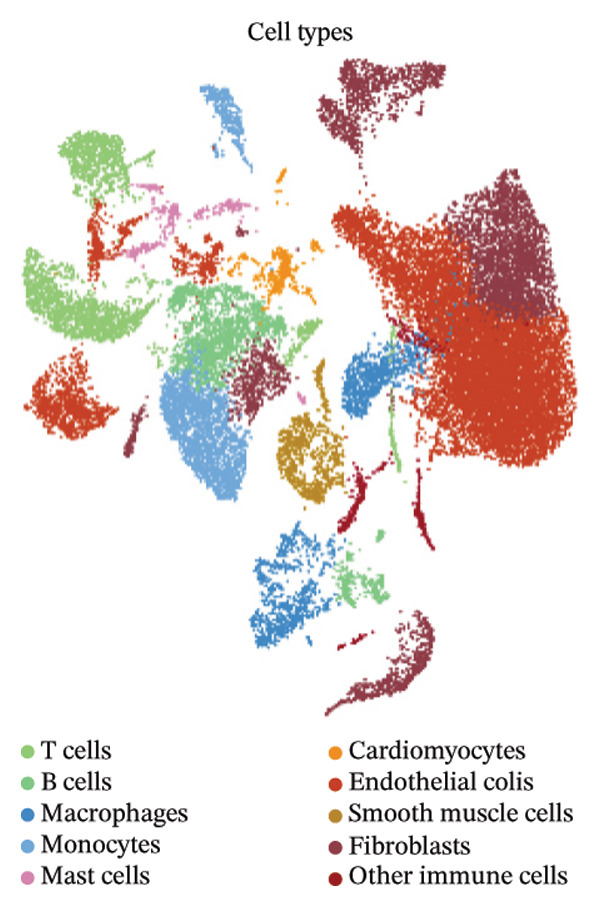
(e)

**Figure:**
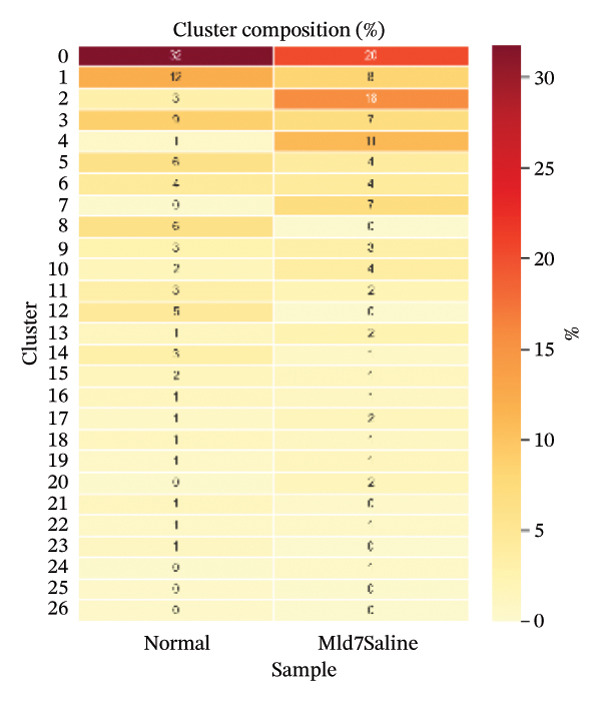
(f)

### 3.3. Canonical Marker Genes Support Accurate Annotation of Immune Subsets

Feature mapping of canonical markers confirmed the reliability of cell type assignment. Cd3e expression localized to T cell regions, while Cd19 was restricted to B cell compartments (Figures 3(a) and 3(b)). Myeloid populations were supported by Csf1r‐enriched macrophage regions and Ly6c2‐enriched monocyte regions, showing distinct spatial patterns consistent with their expected transcriptional identities (Figures 3(c) and 3(d)). A dot plot summarizing representative markers across annotated populations demonstrated concordant expression specificity and detection frequencies, providing orthogonal support for the final cell type labels (Figure 3(e)). In addition, Csf1r expression distributions across samples were consistent with stable macrophage identity signals, enabling downstream evaluation of injury‐associated transcriptional changes within this compartment (Figure 3(f)).

FIGURE 3Expression patterns of canonical marker genes for major cardiac and immune cell populations. (a) Feature plot showing Cd3e expression, identifying T cell populations. (b) Feature plot showing Cd19 expression, identifying B cell populations. (c) Feature plot showing Csf1r expression, marking macrophages. (d) Feature plot showing Ly6c2 expression, indicating monocyte subsets. (e) Dot plot summarizing normalized expression and percentage of expressing cells for representative marker genes across annotated cell types. (f) Violin plot comparing Csf1r expression levels between normal and MI day 7 samples.

**Figure:**
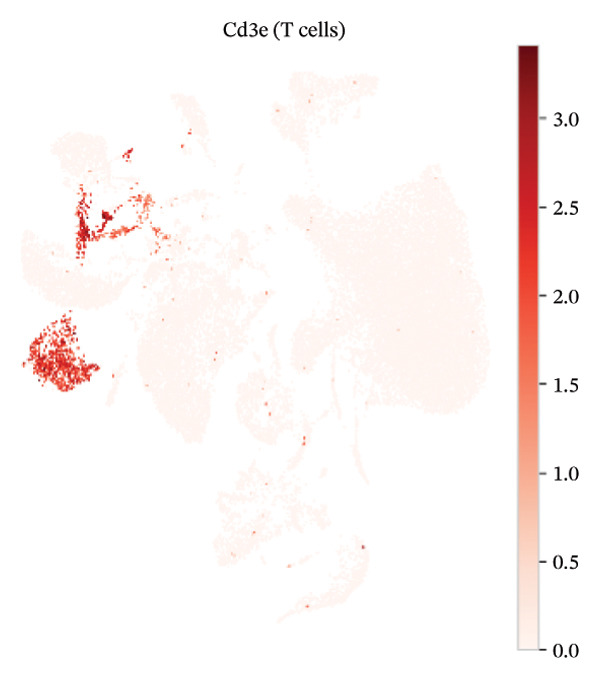
(a)

**Figure:**
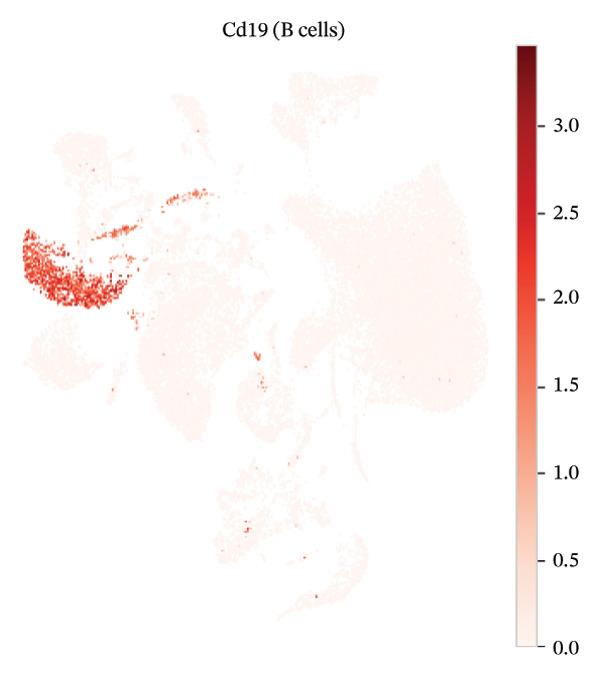
(b)

**Figure:**
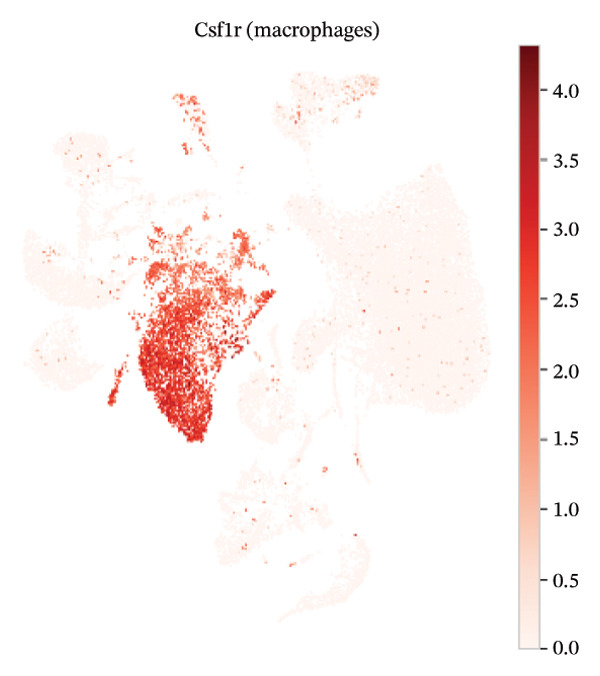
(c)

**Figure:**
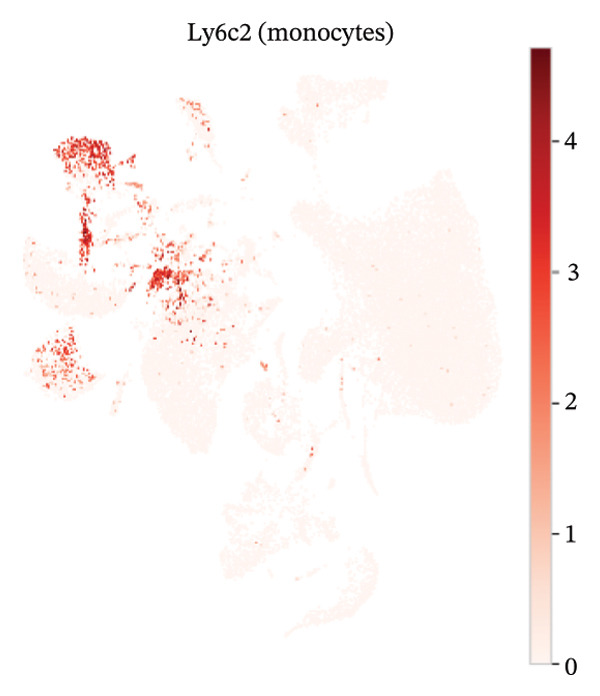
(d)

**Figure:**
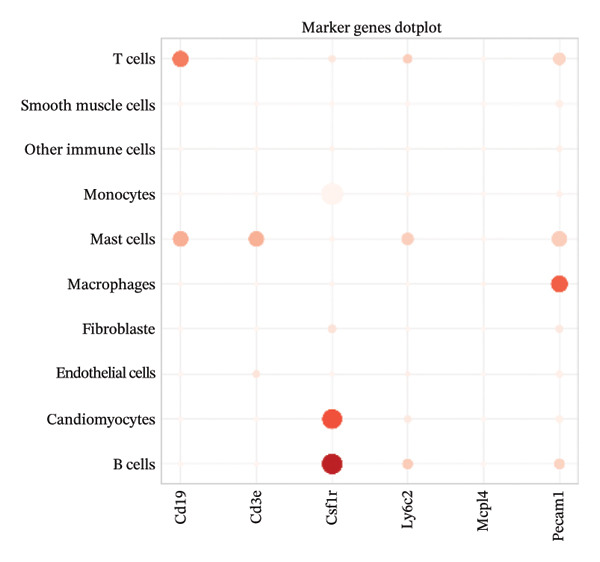
(e)

**Figure:**
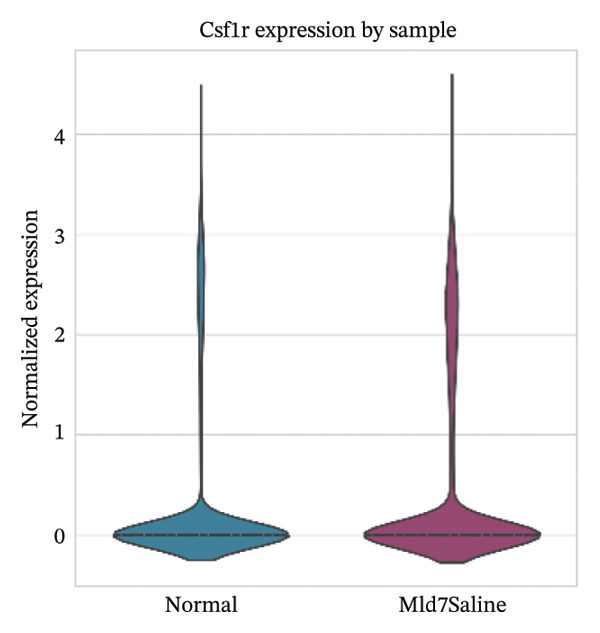
(f)

### 3.4. Differential Expression Analyses Reveal Cell Type–Specific Alterations in Early Transcriptional Programs Following Injury

Differential expression analyses (MI day 7 vs. normal) were then performed within each major cell type to characterize injury‐associated transcriptional changes. Impressive numbers of differentially expressed genes detected in both macrophages (107 upregulated; 81 downregulated) and T cells (71 upregulated; 53 downregulated) reveal tremendous state remodeling in innate versus adaptive immune compartments by volcano plots (Figures 4(a) and 4(b)). In contrast, monocytes showed comparably fewer alterations (13 upregulated; 8 downregulated), indicative of either more modest transcriptional deviations or increased heterogeneity within groups diluting differential signals (Figure 4(c)). Endothelial cells showed limited but detectable changes (17 upregulated; 7 downregulated), while fibroblasts demonstrated extensive transcriptional reorganization with an abundance of upregulated genes (119 upregulated; 18 downregulated), in accordance with injury‐associated stromal activation (Figures 4(d) and 4(e)). B cells showed a strong bias toward downregulation (68 upregulated; 122 downregulated), indicating extensive transcriptional remodeling in this compartment on Day 7 post‐injury (Figure 4(f)). Collectively, these findings suggest that myocardial injury activates strikingly cell type–specific transcriptional responses, resulting in distinctive remodeling of macrophages, T cells, fibroblasts, and B cells.

FIGURE 4Differential gene expression analysis across major cell populations in myocardial injury. (a) Volcano plot showing differentially expressed genes in macrophages (MI day 7 vs. normal). Red dots represent upregulated genes, blue dots represent downregulated genes, and gray dots indicate nonsignificant genes (Up: 107; Down: 81). (b) Volcano plot of differentially expressed genes in T cells (Up: 71; Down: 53). (c) Volcano plot of differentially expressed genes in monocytes (Up: 13; Down: 8). (d) Volcano plot of differentially expressed genes in endothelial cells (Up: 17; Down: 7). (e) Volcano plot of differentially expressed genes in fibroblasts (Up: 119; Down: 18). (f) Volcano plot of differentially expressed genes in B cells (Up: 68; Down: 122).

**Figure:**
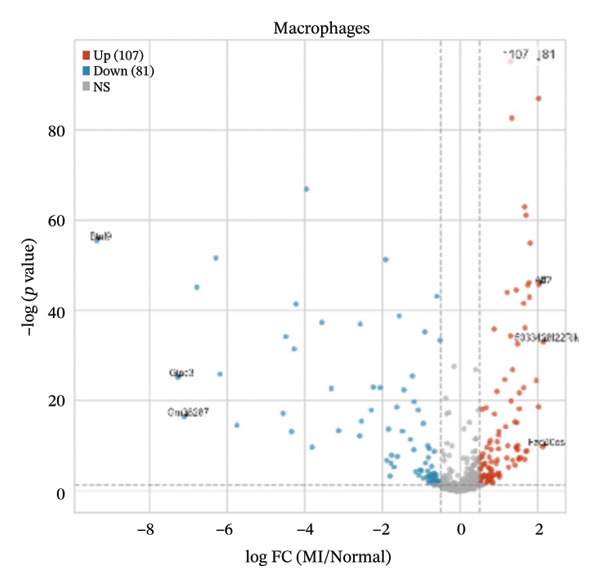
(a)

**Figure:**
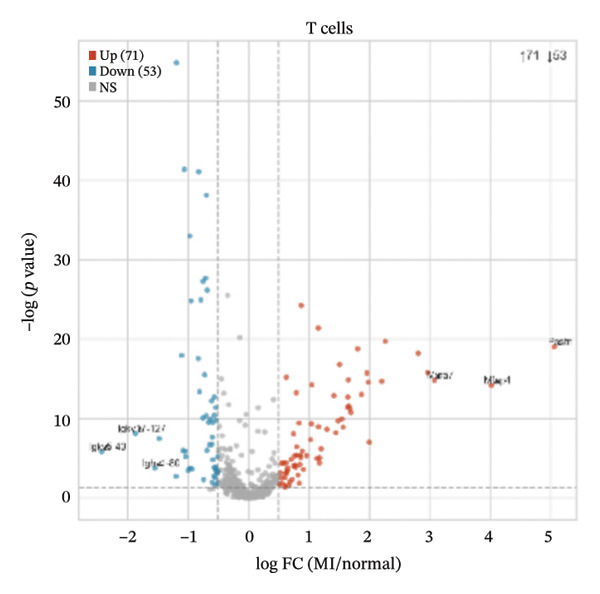
(b)

**Figure:**
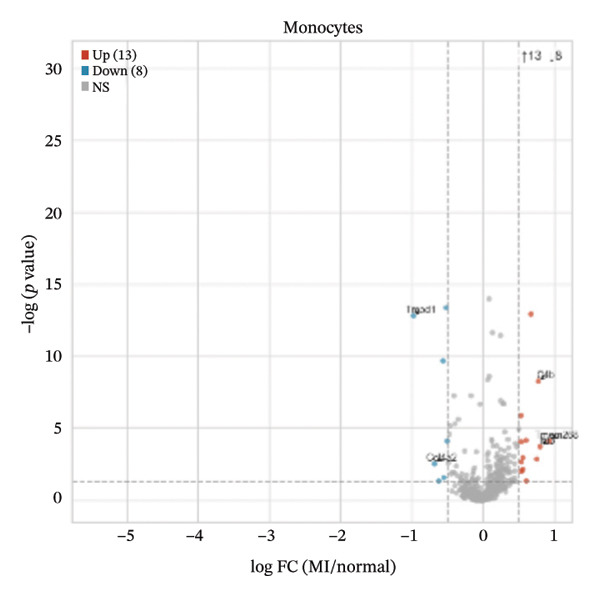
(c)

**Figure:**
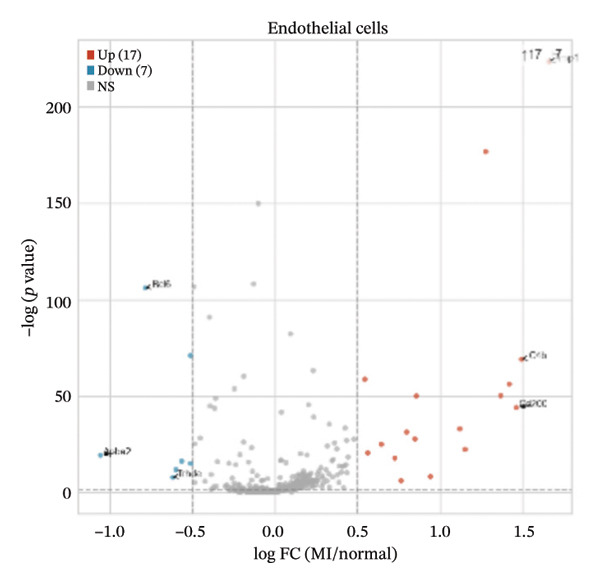
(d)

**Figure:**
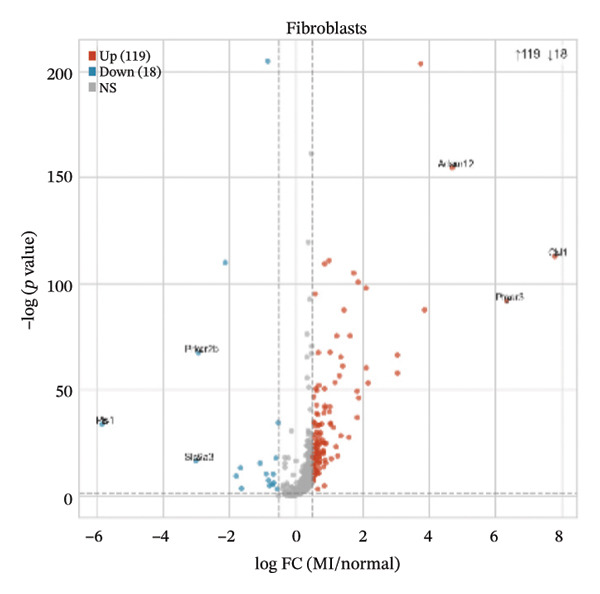
(e)

**Figure:**
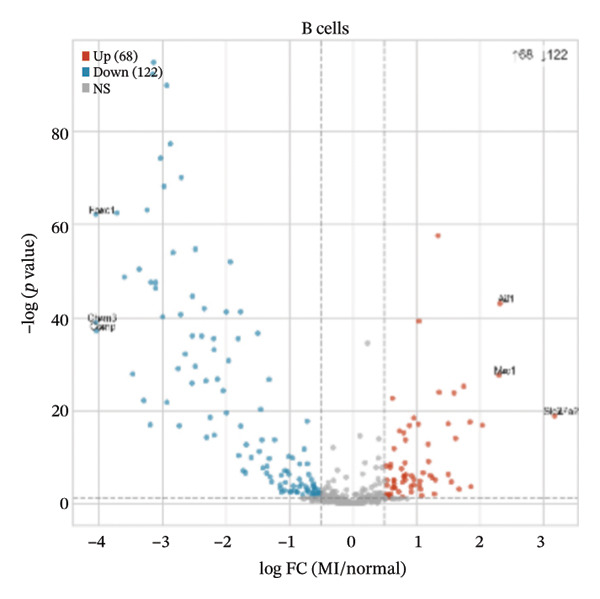
(f)

### 3.5. UMAP Unveils Global Immune Landscape and Spatial Cytokine Signatures

UMAP embedding identified multiple cardiac and immune populations, which exhibited distinct segregation between stromal/cardiac compartments (e.g., fibroblasts, endothelial cells, and cardiomyocytes) and immune subsets (e.g., T cells, B cells, and monocytes/macrophages). Feature mapping confirmed that Tnf expression was localized in distinct immune regions, rather than broadly across all clusters, while Il10 had a sparse, punctate expression with fewer high‐expressing cells. When colored by condition, normal and MI day 7 cells were intermingled in several regions but also showed condition‐enriched areas, indicating myocardial injury–associated remodeling of cellular composition and inflammatory transcriptional states (Figures 5(a)–5(f)).

FIGURE 5UMAP‐based visualization of immune cell annotation and TNF‐α/IL‐10 expression patterns. (a) UMAP plot showing major annotated cell types, including T cells, B cells, monocytes, macrophages, mast cells, endothelial cells, fibroblasts, smooth muscle cells, cardiomyocytes, and other immune cells. (b, c) Feature plots showing the global expression distribution of Tnf (TNF‐α) and Il10 (IL‐10) across all cells. (d) UMAP plot colored by sample group (normal vs. MI day 7 saline, MId7Saline). (e) Feature plot showing Tnf expression in the MI day 7 group. (f) Feature plot showing Il10 expression in the normal group.

**Figure:**
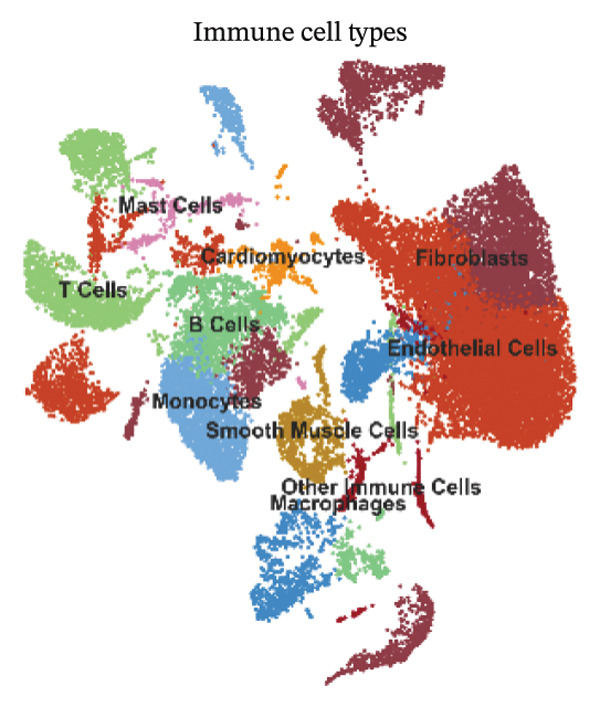
(a)

**Figure:**
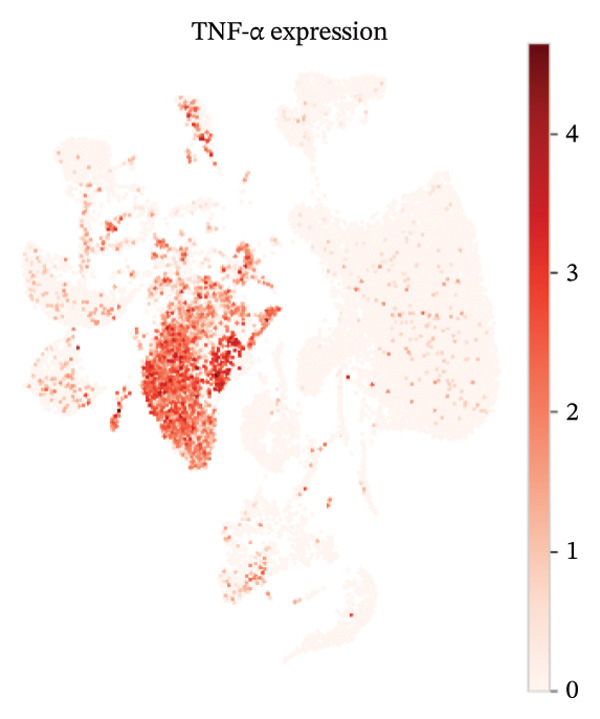
(b)

**Figure:**
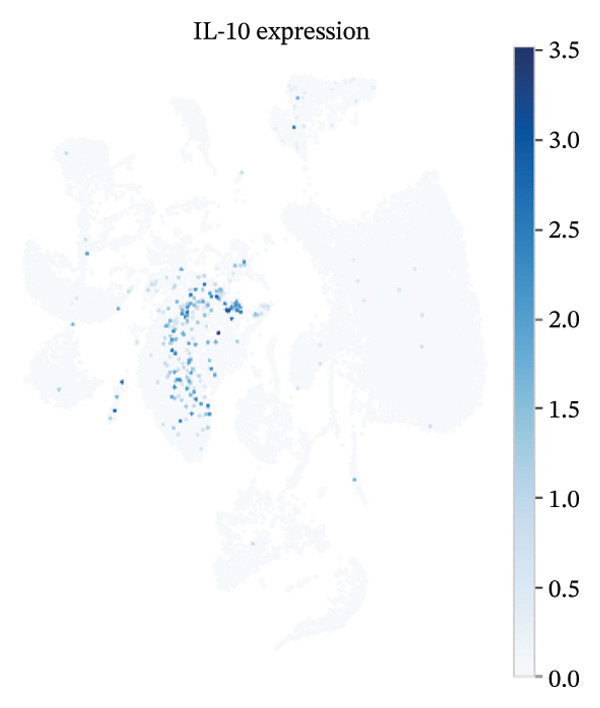
(c)

**Figure:**
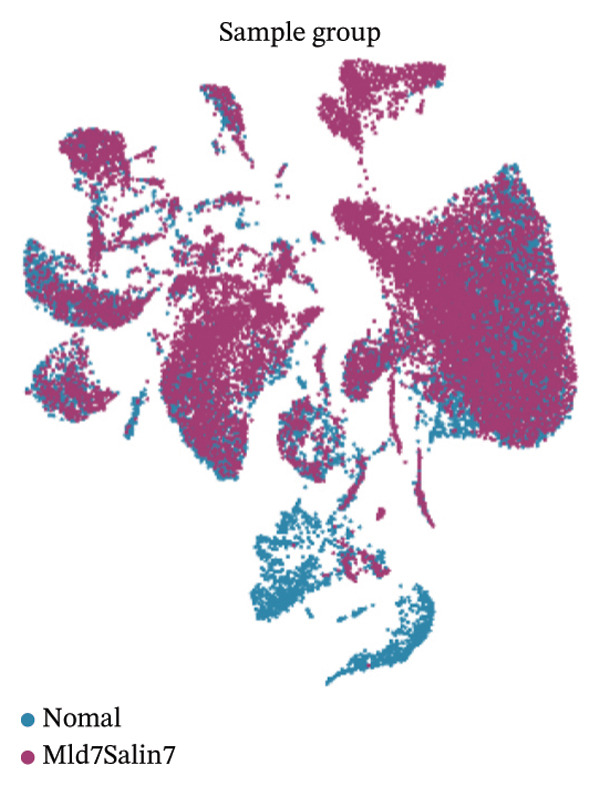
(d)

**Figure:**
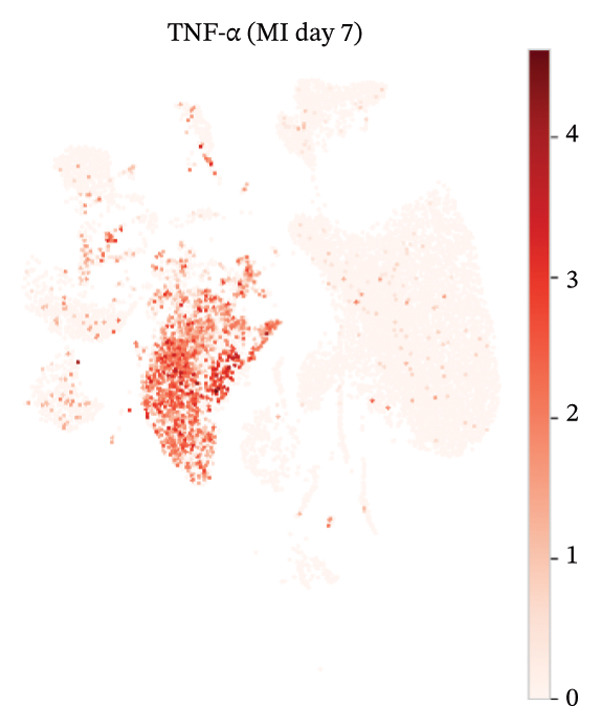
(e)

**Figure:**
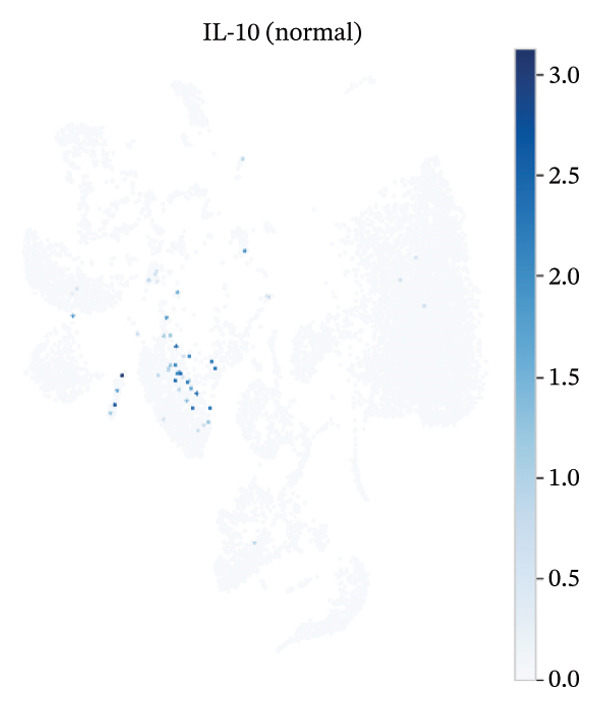
(f)

### 3.6. Myocardial Injury Is Associated With Shifts in Immune Cell Composition

Cell proportion analysis revealed a notable redistribution of immune subsets from normal to MI day 7. In the highlighted immune compartments, B cells increased markedly (approximately ∼2% in normal to ∼12% in MI day 7), while macrophages decreased (approximately ∼9% to ∼3%). Monocytes showed a moderate decrease (approximately ∼10% to ∼8%), whereas T cells displayed a mild increase (approximately ∼8% to ∼9–10%). These compositional changes suggest injury‐associated immune remodeling characterized by expansion of B cells and relative reduction of myeloid macrophage representation at Day 7 post‐injury (Figures 6(a)–6(f)).

FIGURE 6Changes in immune cell proportions between normal and MI day 7 samples. (a) Bar plot showing cell type proportions in the normal sample. (b) Bar plot showing cell type proportions in the MI day 7 (MId7Saline) sample. (c) Grouped bar plot highlighting key immune populations (T cells, B cells, macrophages, and monocytes) across conditions. (d) Comparison of macrophage proportion between normal and MI day 7. (e) Comparison of monocyte proportion between normal and MI day 7. (f) Stacked bar plot summarizing major cell type composition across conditions.

**Figure:**
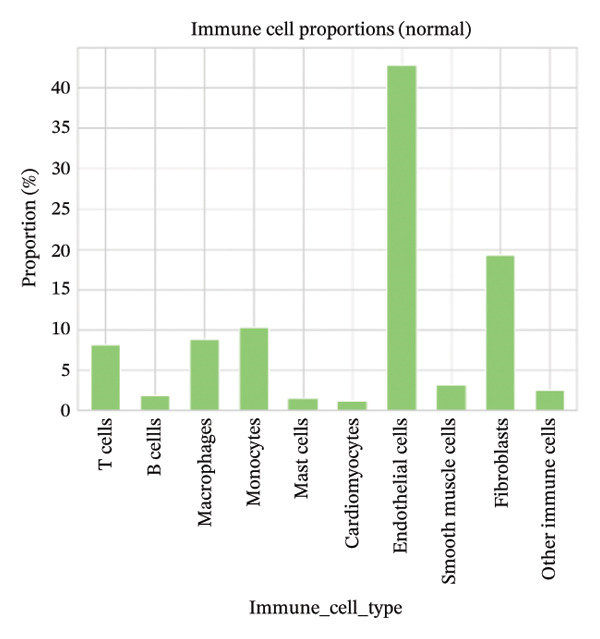
(a)

**Figure:**
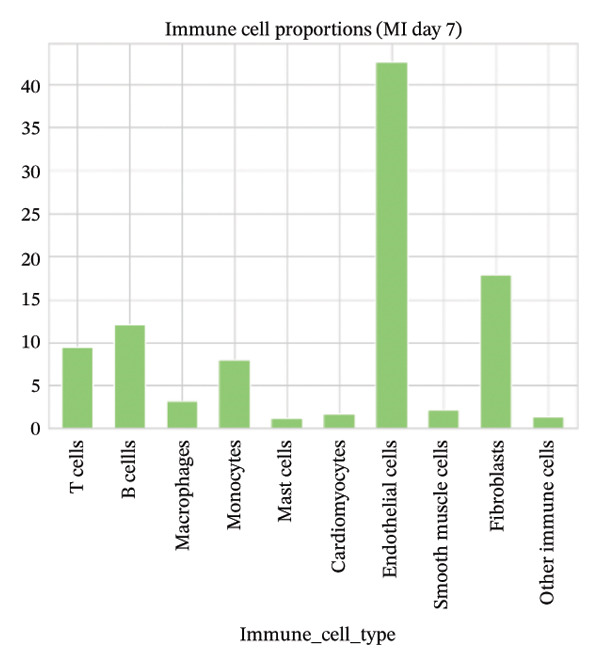
(b)

**Figure:**
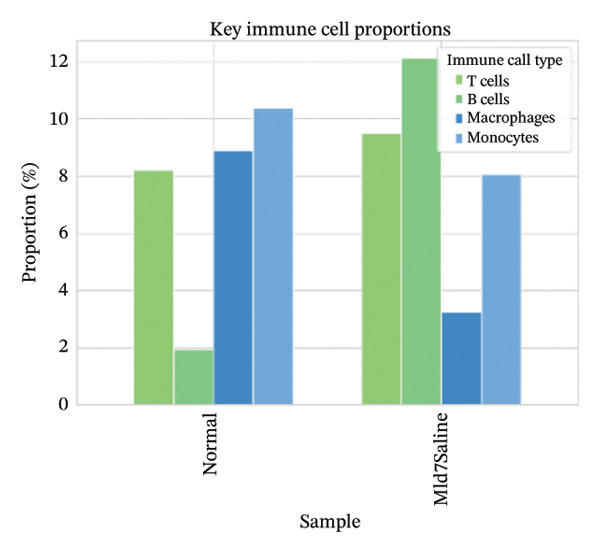
(c)

**Figure:**
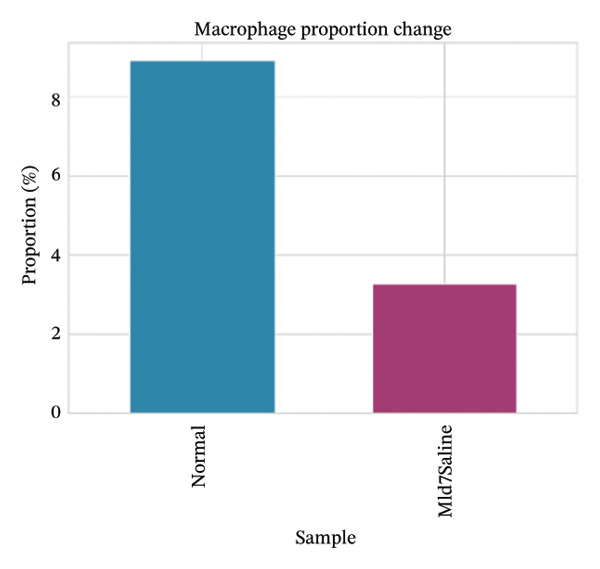
(d)

**Figure:**
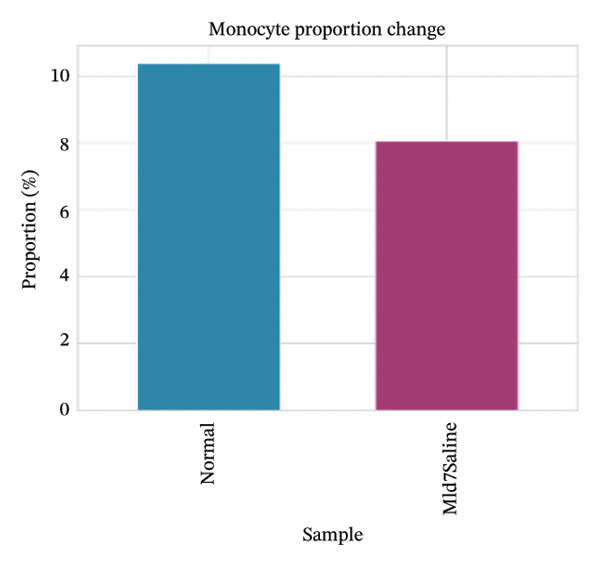
(e)

**Figure:**
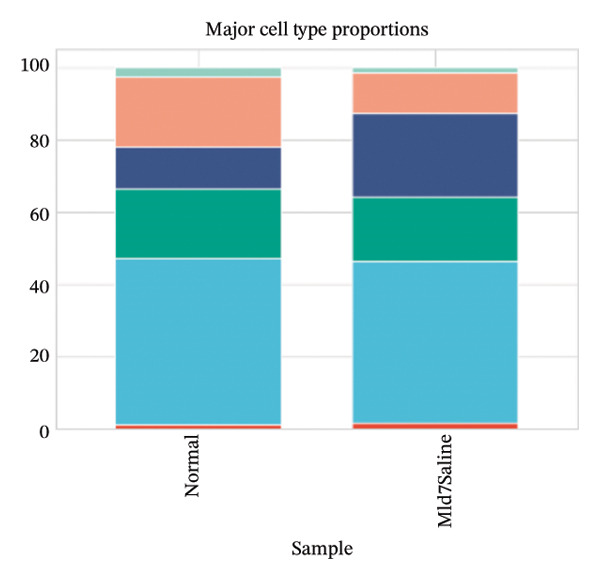
(f)

### 3.7. Cytokine Expression Is Dominated by TNF‐α in Myeloid Compartments and Sparse IL‐10

For Tnf expression across immune subsets, myeloid populations notably had broader and higher‐amplitude distributions, with the strongest signal in monocytes but comparably weaker signals overall in macrophages and lymphocytes, whereas in all immune cell types we examined, Il10 expression was low and infrequently detected, as only a small percentage of the measured cells showed detectable expression. These distributions combined imply that the post‐injury inflammatory signaling at Day 7 is dominantly represented by myeloid transcriptional activities enriched for Tnf, and Il10 expression is limited to vanishingly rare cells (Figures 7(a)–7(f)).

FIGURE 7Violin plots of TNF‐α and IL‐10 expression across immune cell subsets. Violin plots compare the distribution of Tnf (TNF‐α) and Il10 (IL‐10) expression across major immune populations: (a) T cells, (b) B cells, (c) macrophages, (d) monocytes, (e) mast cells, and (f) other immune cells. Distributions are shown for normal and MI day 7 groups.

**Figure:**
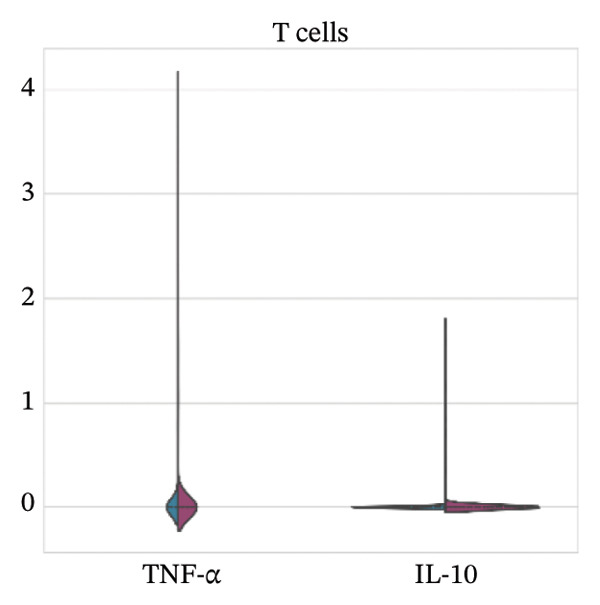
(a)

**Figure:**
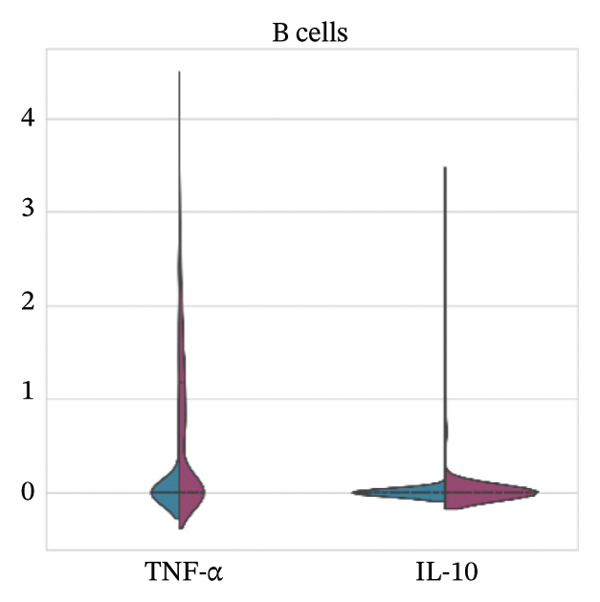
(b)

**Figure:**
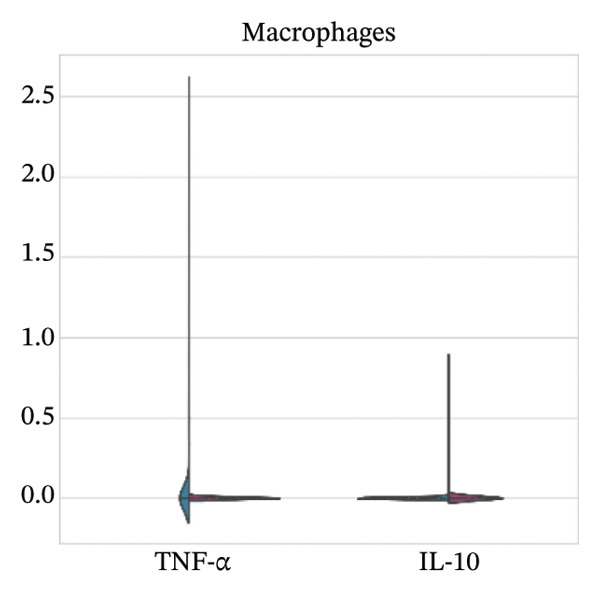
(c)

**Figure:**
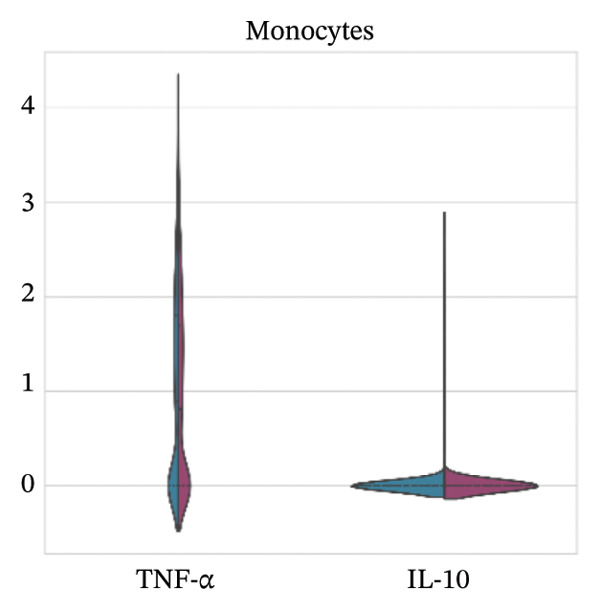
(d)

**Figure:**
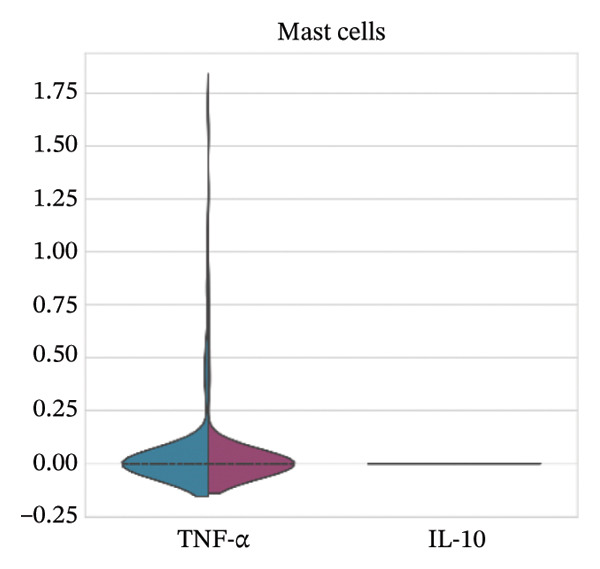
(e)

**Figure:**
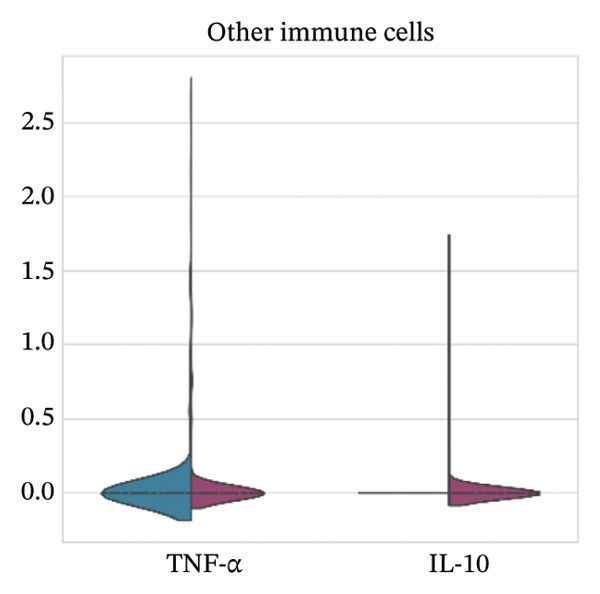
(f)

### 3.8. Macrophage Cytokine Programs Show Limited IL‐10 and Weak TNF‐α With Minimal Coexpression

Within macrophages, UMAP visualization revealed two major macrophage regions with partial condition mixing, suggesting shared transcriptional states alongside condition‐skewed density. Feature plots showed limited Tnf expression confined to a small subset of macrophages and rare Il10‐positive macrophages. Consistently, TNF‐α/IL‐10 scatter plots indicated minimal coexpression, with most cells near zero for Il10 and only a few cells showing elevated cytokine signals. The summary heatmap further supported that strong Tnf signals are more prominent in monocytes (highest mean values across conditions) than in macrophages, whereas macrophage mean expression values remained low overall (Figures 8(a)–8(f)).

FIGURE 8Macrophage‐focused UMAP and TNF‐α/IL‐10 coexpression patterns. (a) UMAP of macrophages colored by sample group (normal vs. MI day 7). (b, c) Feature plots of Tnf and Il10 expression within macrophages. (d) Scatter plot of Tnf vs. Il10 expression in the normal condition. (e) Scatter plot of Tnf vs. Il10 expression in the MI day 7 condition. (f) Heatmap summarizing mean expression of Tnf and Il10 across selected immune cell types and conditions.

**Figure:**
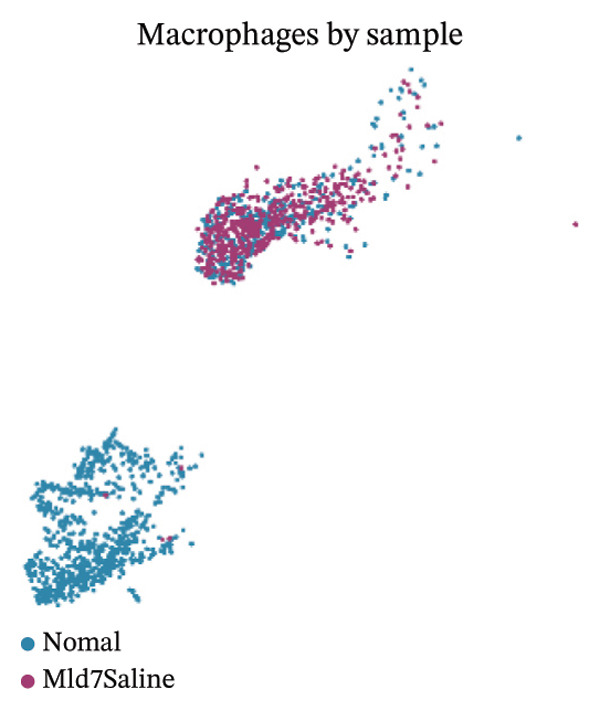
(a)

**Figure:**
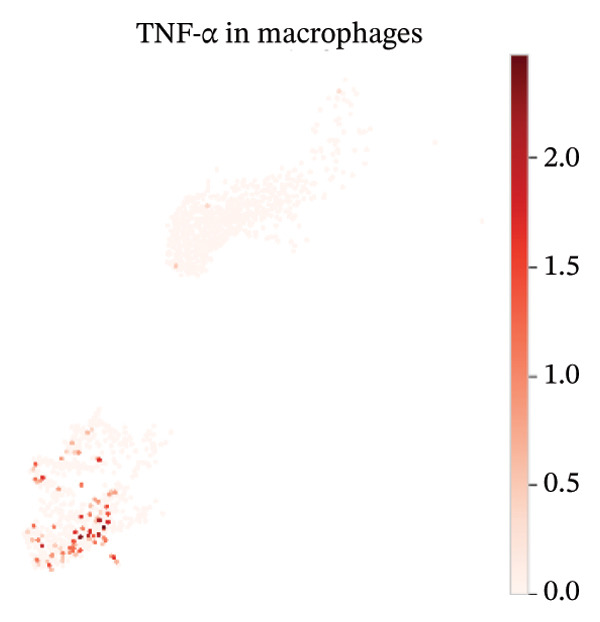
(b)

**Figure:**
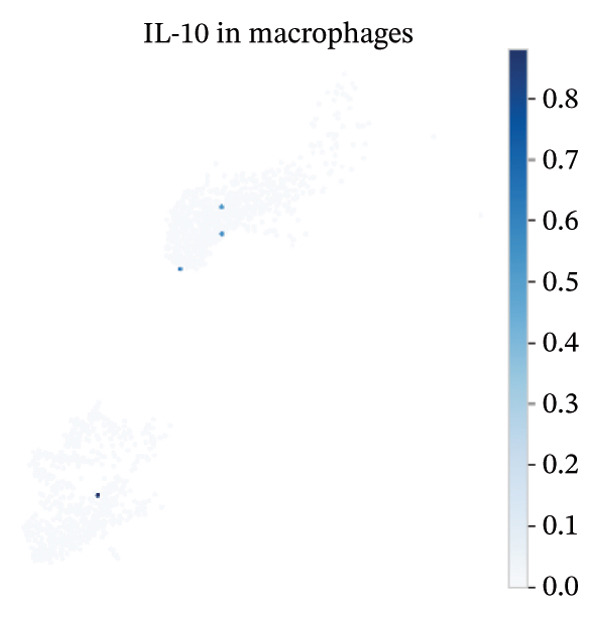
(c)

**Figure:**
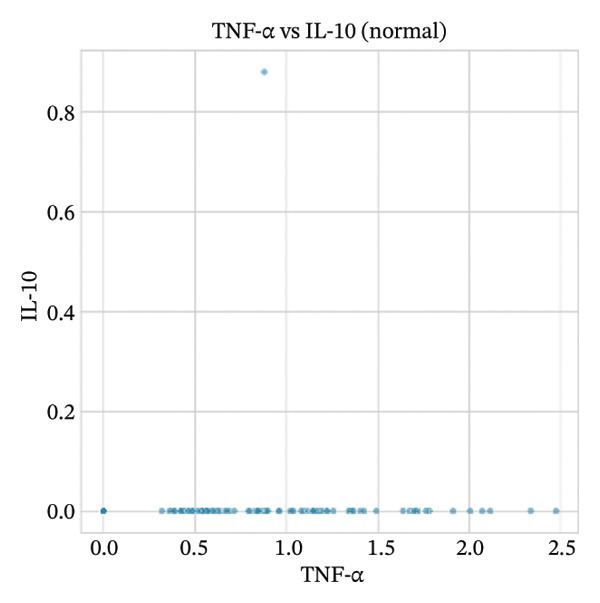
(d)

**Figure:**
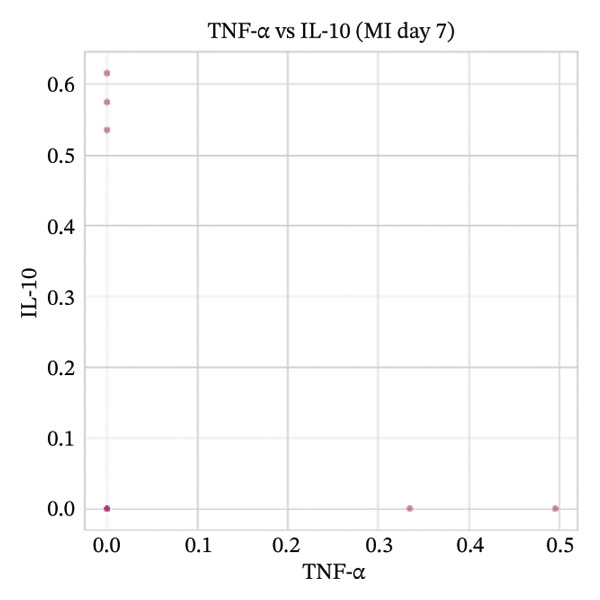
(e)

**Figure:**
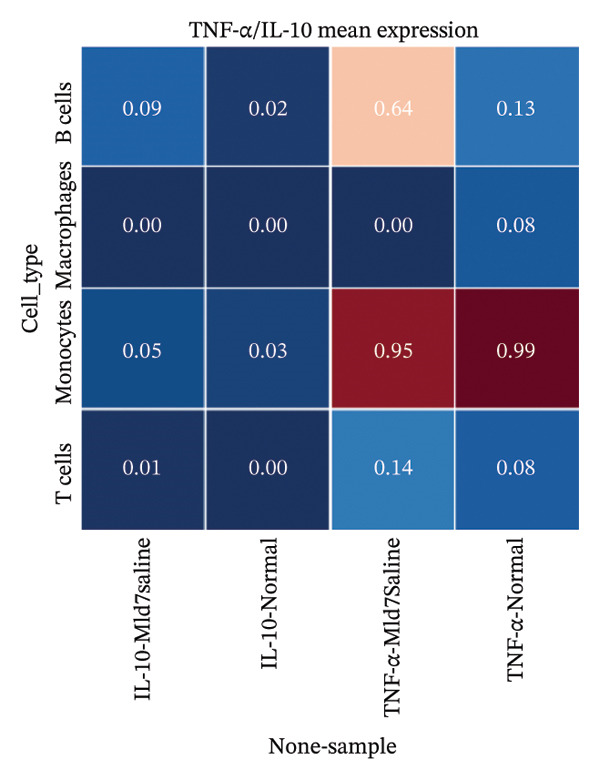
(f)

### 3.9. MR1‐Dependent Regulation of Macrophage Cytokine Expression by Activated MAIT Cells

**FIGURE 9:**
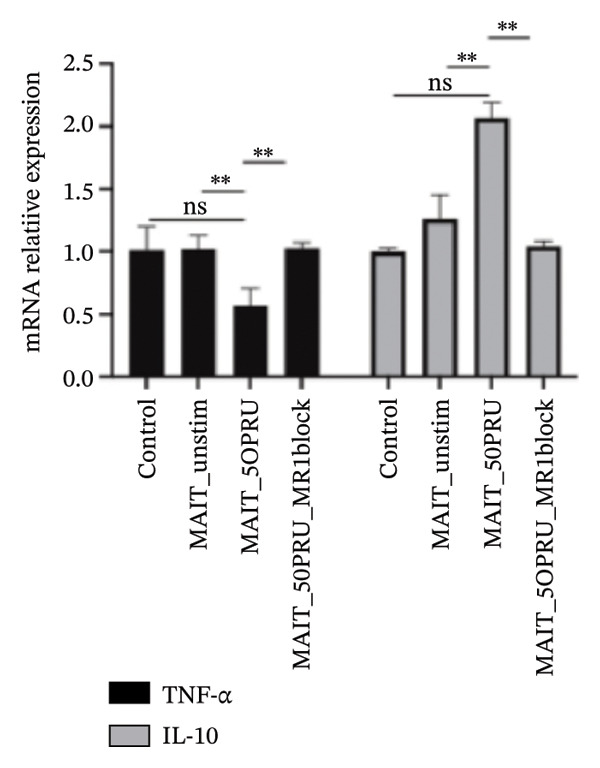
Effects of MAIT cell activation on macrophage cytokine expression. Relative mRNA levels of TNF‐α and IL‐10 in macrophages were determined by qPCR after 24 h of coculture. Experimental groups included control (macrophages alone), MAIT_unstim (macrophages with resting MAIT cells), MAIT_5OPRU (macrophages with 5‐OP‐RU–activated MAIT cells), and MAIT_5OPRU_MR1block (macrophages with 5‐OP‐RU–activated MAIT cells in the presence of MR1 blocking antibody). Data are shown as mean ± SD of three independent experiments. *p* < 0.01. Abbreviation: ns, not significant.

To evaluate the functional changes of macrophage activation at the cellular level, this study also analyzed the expression of pro‐ and anti‐inflammatory cytokines in macrophages under different coculture conditions. qPCR results showed that the baseline expression of TNF‐α and IL‐10 in macrophages alone or in combination with unstimulated MAIT cells remained relatively stable, indicating that unconditioned coculture without antigenic stimulation did not significantly affect cytokine production by macrophages. Upon stimulation with 5‐OP‐RU, however, there was a marked reduction in TNF‐α expression from macrophages combined with a concomitant increase in IL‐10 levels, reflecting an apparent shift toward an anti‐inflammatory phenotype. Significantly, blockade of MR1 signaling largely reversed these effects, restoring cytokine expression back to baseline values. These results confirm the MR1 dependent antigen presentation of MAIT cells resulting in regulation of macrophage cytokine production (Figure 9).

## 4. Discussion

Single‐cell analysis shows MAIT cells undergo extensive transcriptional reprogramming after myocardial injury, with increased expression of activation markers and functional diversification into subsets. These findings of the upregulation of KLRB1 and NCR3 in injured tissues indicate increased immune surveillance capacity and/or cytotoxic functions [5]. The bimodal expression profiles of multiple genes suggest the potential for tissue‐protective and proinflammatory functional specialization among MAIT cell subsets.

Core MAIT cell identity genes like ZBTB16 and SLC4A10 are stably expressed under baseline conditions but dynamically regulated during injury, reflective of the importance these transcriptional programs play in maintaining cellular identity while allowing plasticity of function [15, 16]. As the master transcriptional regulator of MAIT cells, ZBTB16 likely orchestrates the expression of effector programs that allow fast responses to tissue damage cues [17]. Persistence of IL18R1 expression indicates that cells maintain responsiveness to IL‐18, a critical damage‐associated molecular pattern due to tissue injury leading to immune activation [18, 19].

By describing the dynamics of macrophage polarization across diverse populations, this study goes beyond the traditional M1/M2 paradigm. The heterogeneous expression of Mrc1, Arg1, and Nos2 across experimental conditions indicates that macrophage functional states are better placed along a continuum than at distinct polarization states [20]. This finding aligns with the recently emerging concepts of macrophage plasticity and context‐dependent activation. Consistent with this, our coculture studies also demonstrated that activated MAIT cells successfully inhibited TNF‐α and upregulated IL‐10 in macrophages; all displayed the switch of transition to an anti‐inflammatory phenotype on a MR1‐reliant basis. This finding aligns well with novel concepts of macrophage plasticity and context‐specific activation. Consistently, our coculture experiments further confirmed that activated MAIT cells were able to induce suppression of TNF‐α and upregulation of IL‐10 in macrophages; both underwent a MR1‐dependent transition to an anti‐inflammatory phenotype. The universal expression of Cd68 in every sample confirms the accuracy of macrophage identification [21], whereas the disparate expression polarity markers reflected substantial functional heterogeneity among macrophage populations. This differential expression of Tnf and Ccl2 in spatial gradients might imply that local microenvironmental cues, such as proximity to injured cardiomyocytes, vascular structures, or other immune cells, may regulate macrophage activation states [22, 23].

Cellular organization by UMAP clustering of immune marker genes highlights functional interactions across diverse immune populations. The presence of specific cell types and the gradient distributions of chemokines such as Ccl2 suggest the distal recruitment and placement within the injured myocardium [24]. Such spatial relationships are likely reflective of orchestrated immune responses including MAIT cells, macrophages, and conventional T and B cells.

The high expression of cytotoxic molecules, PRF1 and GZMA, in certain cellular compartments suggests active immune effector functions that may lead to both pathogen clearance as well as tissue damage [25, 26]. The balance between protective and pathologic immune responses is likely dictated by the spatiotemporal dynamics of these cellular interactions during the injury–repair continuum.

These data define specific gene expression signatures within MAIT cells and macrophage subpopulations, which may offer potential biomarkers for monitoring immune activity in cardiovascular disease [27]. Existing temporal expression patterns of important generalist regulatory genes like ZBTB16 and KLRB1 and macrophage polarization markers may present targets for therapeutic intervention toward immune modulation resulting in effective tissue healing versus inflammatory overkill [28]. These findings provide experimental support for the concept that altering MAIT macrophage interactions may be a novel approach to modulating proinflammatory states in myocardial injury.

Further studies are needed to validate these observations in human cardiac injury models and investigate how MAIT cell activation influences the processes of cardiac repair. Furthermore, advancements in targeted therapies that modulate MAIT cell functions or direct the polarization of macrophages toward tissue‐protective phenotypes can open an exciting new therapeutic avenue to improve outcomes in myocardial injury and other forms of cardiovascular pathology.

## 5. Limitations

Although this study offers important information regarding immune cell dynamics in injured myocardium, limitations must be acknowledged. Using publicly available datasets can cause batch effects and experimental variabilities that could affect the outputs’ interpretation. In the absence of longitudinal approaches linking injury with repair, our understanding of temporal dynamics and cellular fate transition through the injury–repair continuum is also limited due to the cross‐sectional nature of single‐cell snapshots. In addition, while our additional in vitro experiments demonstrated that activated MAIT cells modulate cytokine expression by macrophages in an MR1‐dependent manner, these results were established using immortalized cell line–based models and need to be validated in primary human specimens and in vivo experimental systems.

## 6. Conclusions

This comprehensive single‐cell analysis demonstrates significant transcriptional reprogramming of MAIT cells and complex polarization dynamics of macrophage populations during myocardial injury. The identification of specific gene expression signatures and cellular interaction patterns provides new insights into immune mechanisms underlying cardiac pathophysiology. In addition, our in vitro validation experiments showed that activated MAIT cells can downregulate TNF‐α and enhance IL‐10 expression in macrophages in an MR1‐dependent manner, supporting the translational relevance of these single‐cell observations.

## Funding

This work was supported by the Wenzhou Science and Technology Bureau (grant number: Y20240241) and the Joint TCM Science & Technology Projects of National Demonstration Zones for Comprehensive TCM Reform (grant number: 2026ZL0825).

## Ethics Statement

The authors have nothing to report.

## Conflicts of Interest

The authors declare no conflicts of interest.

## Data Availability

The data that support the findings of this study are available from the corresponding authors upon reasonable request.
